# Fine Mapping of a Clubroot Resistance Gene in Chinese Cabbage Using SNP Markers Identified from Bulked Segregant RNA Sequencing

**DOI:** 10.3389/fpls.2017.01448

**Published:** 2017-08-28

**Authors:** Zhen Huang, Gary Peng, Xunjia Liu, Abhinandan Deora, Kevin C. Falk, Bruce D. Gossen, Mary R. McDonald, Fengqun Yu

**Affiliations:** ^1^Saskatoon Research and Development Centre, Agriculture and Agri-Food Canada, Saskatoon SK, Canada; ^2^State Key Laboratory of Crop Stress Biology for Arid Areas, College of Agronomy, Northwest A&F University Yangling, China; ^3^Department of Plant Agriculture, University of Guelph, Guelph ON, Canada

**Keywords:** clubroot, RNA-Seq, SNPs, fine mapping, *Brassica rapa*, *Plasmodiophora brassicae*

## Abstract

Clubroot, caused by *Plasmodiophora brassicae*, is an important disease of canola (*Brassica napus*) in western Canada and worldwide. In this study, a clubroot resistance gene (*Rcr2*) was identified and fine mapped in Chinese cabbage cv. “Jazz” using single-nucleotide polymorphisms (SNP) markers identified from bulked segregant RNA sequencing (BSR-Seq) and molecular markers were developed for use in marker assisted selection. In total, 203.9 million raw reads were generated from one pooled resistant (R) and one pooled susceptible (S) sample, and >173,000 polymorphic SNP sites were identified between the R and S samples. One significant peak was observed between 22 and 26 Mb of chromosome A03, which had been predicted by BSR-Seq to contain the causal gene *Rcr2*. There were 490 polymorphic SNP sites identified in the region. A segregating population consisting of 675 plants was analyzed with 15 SNP sites in the region using the Kompetitive Allele Specific PCR method, and *Rcr2* was fine mapped between two SNP markers, SNP_A03_32 and SNP_A03_67 with 0.1 and 0.3 cM from *Rcr2*, respectively. Five SNP markers co-segregated with *Rcr2* in this region. Variants were identified in 14 of 36 genes annotated in the *Rcr2* target region. The numbers of poly variants differed among the genes. Four genes encode TIR-NBS-LRR proteins and two of them *Bra019410* and *Bra019413*, had high numbers of polymorphic variants and so are the most likely candidates of *Rcr2*.

## Introduction

*Plasmodiophora brassicae* Woronin is a biotrophic soil-borne pathogen in the Infrakingdom Rhizaria (Nikolaev et al., 2004) that causes clubroot disease in Brassica oil and vegetable crops. It produces long-lived resting spores that are difficult to control using standard strategies such as anti-microbial compounds or crop rotations (Voorrips, 1995; Tsushima, 2000). Therefore, genetic resistance is the most effective approach to manage this disease.

Clubroot is an increasing problem on canola (*Brassica napus*) in western Canada and worldwide. Sources of clubroot resistance in canola are limited, but genotypes with resistance to a broad range of pathotypes of *P. brassicae* have been identified in the canola progenitor species *B. rapa* (Hasan et al., 2012; Peng et al., 2014). These lines could be used to broaden the genetic base of clubroot resistance in canola (Yu et al., 2016). Introgression of important agronomic traits such as clubroot resistance from *B. rapa* into canola is possible through conventional breeding (Yu et al., 2012; Gautam et al., 2013; Li et al., 2013), but it is important to identify and map the resistance genes in *B. rapa* so that genes can be transferred into canola efficiently.

More than 10 genes conferring resistance to clubroot have been identified in vegetable cultivars of *B. rapa* (Kuginuki et al., 1997; Matsumoto et al., 1998, 2012; Suwabe et al., 2003, 2006; Hirai et al., 2004; Piao et al., 2004, 2014; Saito et al., 2006; Sakamoto et al., 2008; Cho et al., 2012; Chen et al., 2013; Kato et al., 2013; Peng et al., 2014; Zhang et al., 2014). For example, resistance genes *Crr1, Crr2*, and *Crr4* derived from the European turnip cv. “Siloga” were mapped onto *B. rapa* chromosomes A08, A01, and A06, respectively (Suwabe et al., 2003, 2006). *Crr3* from turnip cv. “Milan White” was mapped onto A03 (Hirai et al., 2004; Saito et al., 2006), while *CRk* and *CRc* from turnip cv. “Debra” were mapped onto A03 and A02, respectively (Sakamoto et al., 2008; Matsumoto et al., 2012). *CRa* from turnip line ECD02 (Matsumoto et al., 1998) and *CRb* from turnip line ECD01 (Piao et al., 2004) were also mapped to A03. A clubroot resistance gene, *Rcr1*, identified in pak choy cv. “Flower Nabana” (*B. rapa*) was also mapped to A03 (Chu et al., 2014; Yu et al., 2016). Three resistance genes *Rcr4, Rcr8*, and *Rcr9* from *B. rapa* canola breeding line T19 were mapped to A03, A02, and A08, respectively through genotyping by sequencing (Yu et al., 2017). Two genes, *Crr1* and *CRa*, have been cloned that encode Toll-Interleukin-1 receptor/nucleotide binding site/leucine-rich repeat (TIR-NBS-LRR, TNL) proteins (Ueno et al., 2012; Hatakeyama et al., 2013).

Genetic mapping is a powerful approach for identification of causal genes associated with economically important traits and development of molecular markers tightly linked to the traits for marker assisted selection in plant breeding. Several types of molecular markers, such as restriction fragment length polymorphism, microsatellite or simple sequence repeats, and single-nucleotide polymorphisms (SNP) have been employed in genetic mapping. However, discovery of the molecular markers through conventional approaches is very time consuming, tedious, and expensive. Next-generation sequencing (NGS) for genetic mapping has facilitated the rapid development and application of genomics tools in plant breeding (Bolger et al., 2014). NGS has been used for genetic mapping. An important mapping technique, bulked segregant analysis (BSA), consists of two bulks of individual plants with extreme phenotypes, has been utilized—for many years to identify markers linked to major genes of interest, such as those for disease resistance (Michelmore et al., 1991). BSA has also been coupled with RNA sequencing (RNA-Seq) for gene mapping (Liu et al., 2012).

Screening of *Brassica* cultivars for clubroot resistance to the predominant pathotypes of *P. brassicae* identified in Canada was performed. Several *B. rapa* vegetable cultivars, including pak choy cv. “Flower Nabana” and Chinese cabbage (*B. rapa* subsp. *pekinensis*) cv. “Jazz,” were resistant to clubroot (Peng et al., 2014). Also, the clubroot resistance gene *Rcr1* in “Flower Nabana” was identified (Chu et al., 2014). The current study describes the identification and mapping of a clubroot resistance gene from Chinese cabbage cv. “Jazz” using SNP markers identified with bulked segregant RNA-Seq (BSR-Seq).

The objectives of the study were to: (i) determine the inheritance of clubroot resistance in cv. “Jazz”; (ii) map the resistance gene; (iii) develop SNP markers tightly linked to the resistance gene; and (iv) examine DNA variation in the target region and identify the most probable candidate(s) for the gene.

## Materials and Methods

### Plant Materials and Mapping Population

Chinese cabbage cv. “Jazz” (American Takii, Salinas, CA, United States) is a hybrid that is highly resistant to the five main pathotypes of *P. brassicae* found in Canada (Peng et al., 2014). One plant was used to pollinate a doubled-haploid canola-quality line of *B. rapa*, ACDC (Saskatoon Research and Development Centre, Agriculture and Agri-Food Canada) that is self-compatible and highly susceptible to *P. brassicae*. Reciprocal crosses were made between “Jazz” and ACDC to produce F_1_ progenies. The parents and F_1_ plants were tested for resistance to pathotype 3 (Williams, 1966) of *P. brassicae.* Segregation of resistant (R) and susceptible (S) phenotypes in the F_1_ segregating populations was analyzed using Chi-square (χ^2^) tests for goodness of fit (Sokal and Rohlf, 1995).

In addition, F_1_ plants were self-pollinated, but only 46 of 200 plants produced seed because of self-incompatibility. Plants from the 46 self-fertile lines (F_2_) were used to evaluate resistance to additional pathotypes of *P. brassicae*. The F_2_ lines were equivalent to self-pollinated BC_1_ populations (BC_1_S_1_).

### Pathogen Inoculation

A field collection of pathotype 3 of *P. brassicae* was used to inoculate plants for studies on inheritance and genetic mapping. Preparation of inoculum, inoculation and plant rating followed standard protocols (Chu et al., 2014). The clubroot reaction of F_2_ lines to pathotypes 2, 3, 5, 6, and 8 was assessed under controlled conditions (Yu et al., 2016). Clubroot severity on 7-14 plants per line at 6 weeks after inoculation was assessed using a standard 0–3 scale. A disease severity index (DSI; Strelkov et al., 2006) was calculated using the following formula:

$$\begin{array}{c} \mathrm{DSI}(\%)=\frac{\Sigma (\mathrm{rating}\;\mathrm{class})\times (\#\mathrm{plants}\;\mathrm{in}\;\mathrm{rating}\;\mathrm{class})}{(\mathrm{total}\;\#\;\mathrm{plants}\;\mathrm{in}\;\mathrm{treatment})\times 3}\times 100 \end{array}$$

A highly susceptible control, Shanghai pak choy cv. “Mei Qing Choi” (Stokes Seeds, ON, Canada), was included with each group to ensure that inoculation was effective. The evaluation of clubroot reaction to pathotypes was repeated.

Correlation coefficients among the DSI values in F_2_ families to the five pathotypes of *P. brassicae* were calculated using Microsoft Excel. The significance of the correlation coefficients was determined with *t*-tests (Iversen and Gergen, 1997).

### RNA-Seq and Sequence Alignment

The F_1_ population from ACDC (female) × “Jazz” (male) was used for RNA-Seq. At 15 days post-inoculation, leaf tissue from 30 R plants and 30 S plants was combined to form R and S bulks, respectively; together, the two bulks comprised one biological replicate. Three replicates with a total of 90 R and 90 S plants were assessed. RNeasy Plant Mini Kit (Qiagen; Toronto, ON, Canada) with on-column deoxyribonuclease (DNase) digestion was used for RNA extraction from each sample following the manufacturer’s instructions. The RNA concentration and quality were checked using a NanoDrop 2000c Spectrophotometer (Thermo Scientific, Waltham, MA, United States) and an Agilent Bioanalyzer 2100 (Agilent Technologies; Mississauga, CA, United States), to ensure that the RNA integrity number (RIN) was >8 for each sample. The preparation of cDNA libraries, RNA-Seq and DNA alignment were performed at Databio2 LLC (Ames, IA, United States). Prior to alignment, the nucleotides of each raw read were scanned for quality, and bases with PHRED quality value <15 (out of 40) (Ewing and Green, 1998; Ewing et al., 1998) were removed by the trimming pipeline at Databio2 LLC. Trimmed short reads were aligned to the reference genome (*B. rapa*, V1.5^1^) using GSNAP (Wu and Nacu, 2010) as paired-end fragments with ≤2 mismatches. Short reads from the three R bulks were combined and assembled into the reference genome, as were the three S bulks, to produce separate assembly files, pooled R and pooled S.

### SNP Discovery and Mapping of the Causal Gene

SNP discovery and mapping of the causal gene were performed using the uniquely aligned short reads from the two pooled samples with Databio2 LLC. Polymorphisms at each potential SNP site were examined and putative SNPs were identified using the following criteria: (1) the first and last three aligned bases of each read were discarded; (2) each polymorphic base must have a PHRED base quality value of at least 15; (3) at least five unique reads must support the base-pair call. An empirical Bayesian approach was used to estimate the conditional probability of no recombination between each SNP marker and the causal gene in both the R pool and the S pool (Liu et al., 2012). The posterior probability of each SNP indicates the probability of complete linkage between the SNP and causal gene.

### SNP Genotyping and Linkage Analysis

The same genotyping method as that described by Yu et al. (2016) was used in this study. Genomic DNA was extracted from young leaves of F_1_ segregating population consisting of 675 plants (including the 180 plants for RNA-Seq) using the CTAB method (Doyle and Doyle, 1990). Selected SNPs identified in the target region were confirmed using the Kompetitive Allele Specific PCR (KASP) method^2^ following the manufacture’s instruction. PCR reactions were performed using a StepOne Plus Real Time PCR System (Applied Biosystem, Mississauga, ON, Canada). Linkage groups were performed with JoinMap 4.1 (Van Ooijen and Voorrips, 2001).

### Identification of Variants in the Target Region

The software SeqMan NGen 13 (DNASTAR, Madison, WI, United States) was used for short read assembly using the pooled sample assembly method (Yu et al., 2016). Standard assembling and filtering parameters were used. Discovery of SNP and InDel variants from the DNA sequences in *B. rapa* “Chiifu” were performed using software SeqMan Pro 13 (DNASTAR) with Q call ≥15 and depth ≥5.

## Results

### Inheritance of Clubroot Resistance in Chinese Cabbage cv. “Jazz”

**FIGURE 1:**
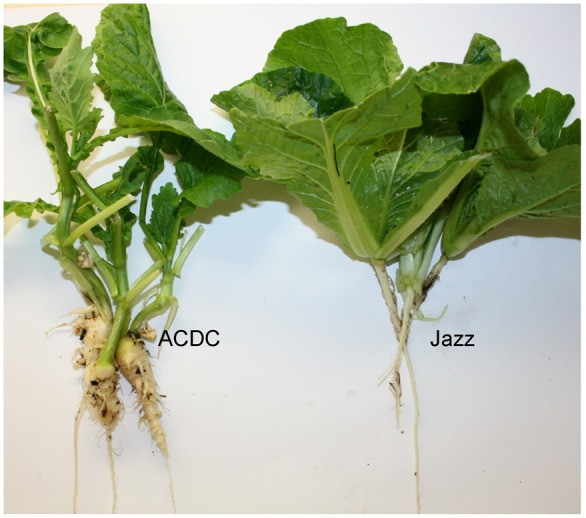
Phenotypes of the *Brassica rapa* parents: Chinese cabbage cv. “Jazz” (R) and susceptible canola breeding line ACDC. Plants were inoculated with pathotype 3 of *Plasmodiophora brassicae*.

Each “Jazz” plant was resistant to pathotype 3 of *P. brassicae* and all of the ACDC plants were susceptible, with characteristic clubroot symptoms at 5 weeks after inoculation (**Figure 1**). Segregation for R and S consistent with an expected ratio of 1:1 was observed in the F_1_ populations derived from the reciprocal crosses (**Table 1**). These results indicated that the resistance in “Jazz” was associated with a single dominant nuclear gene (designated as *Rcr2*) and that “Jazz” was heterozygous at the *Rcr2* locus.

**Table 1:** Genetic analysis of clubroot resistance to pathotype 3 of *Plasmodiophora brassicae* in crosses of *Brassica rapa* cv. “Jazz” with line ACDC.

Parents and crosses	Type	Disease rating (0–3)					Phenotypes and expected ratio (1:1)			
		0	1	2	3	Total	R	S	χ^2^	*P*
Jazz	R parent	23	0	0		23	23	0	
ACDC	S parent	0	0	3	16	19	0	19		
ACDC × Jazz	F_1_	16	1	0	12	29	16	13	0.31	0.58
Jazz × ACDC	F_1_	13	2	1	11	27	13	14	0.04	0.84

*R, resistant; S, susceptible. A rating of 0 was defined as R, 1-3 as S.*

### Resistance to Multiple Pathotypes of*P. brassicae* in F_2_ Lines

“Jazz” was identified previously as highly resistant to several pathotypes (2, 3, 5, 6, and 8) of *P. brassicae* (Peng et al., 2014), but *Rcr2* was identified by testing with pathotype 3 only. To determine if clubroot resistance to pathotypes 2, 5, 6, and 8 in “Jazz” was associated with *Rcr2*, 46 F_2_ lines were tested for resistance to the five pathotypes. “Jazz” was highly resistant (DSI = 0), while both ACDC and the susceptible control (Shanghai pak choy cv. “Mei Qing Choi”) were highly susceptible (DSI = 100). The distributions of DSI values to the five pathotypes among the F_2_ lines were similar (**Supplementary Figure S1**). The clubroot reaction among the pathotypes was significantly correlated (**Table 2**).

**Table 2:** Correlation coefficients of clubroot severity among the 46 F_2_ lines to pathotypes 2, 3, 5, 6, and 8 of *Plasmodiophora brassicae*.

Pathotype	3	5	6	8
2	0.84^∗∗^	0.87^∗∗^	0.93^∗∗^	0.82^∗∗^
3		0.90^∗∗^	0.88^∗∗^	0.84^∗∗^
5			0.88^∗∗^	0.86^∗∗^
6				0.91^∗∗^

*^∗∗^Significance level at P < 0.01.*

### Data Filtering and Short Reads Assembly

A total of 203.9 million raw reads were generated from the R and S pooled samples. About 98% of the reads passed the quality control standard and remained. After trimming, the average length of reads was reduced from 101 bases to 98–99 bases. A total of 86.16% of the trimmed reads from two pooled samples could be mapped to the reference genome (*B. rapa*_sequence_v1.5.fa), and 78.13% of the trimmed reads were unique alignments (**Table 3**).

**Table 3:** Summary of RNA-Seq short reads from the R pooled sample and the S pooled sample in the F_1_ population from crosses of Chinese cabbage cv “Jazz” with the doubled haploid line ACDC.

Short reads	No. of reads (×10^6^)	Total reads (%)
Total raw reads	203.9	
Short reads after trimming	199.8	97.96
Short read alignments	175.6	86.10
Unique alignments (single locus)	159.3	78.13

### Discovery of Polymorphic SNPs

Unique alignments from the pooled R and the pooled S samples were extracted separately for SNP discovery. Reads were mapped to both chromosomes and scaffolds before assigning SNPs. A total of 173,383 polymorphic SNP between the pooled R and pooled S samples were identified with 170,515 SNPs in the 10 chromosomes and 2,866 SNPs in the scaffolds (**Table 4**). The numbers of polymorphic SNP varied among the chromosomes, with strong positive correlation with length of chromosome (*r* = 0.92). This supported a previous observation (Yu et al., 2016) that there were many more polymorphic SNPs on longer chromosomes (A03, A09) than shorter chromosomes (A04, A10).

**Table 4:** Length of chromosomes and number of polymorphic SNPs identified from BSR-Seq.

Chromosome	Chromosome length (Mb)	No. of SNPs
A01	26.78	14,884
A02	26.93	17,030
A03	31.69	25,390
A04	19.25	10,446
A05	25.28	16,801
A06	25.2	15,934
A07	25.87	16,323
A08	20.81	13,627
A09	38.88	27,100
A10	16.39	12,982
Scaffolds	27.9	2,866
Total	285	173,383

### Mapping of *Rcr2*

**FIGURE 2:**
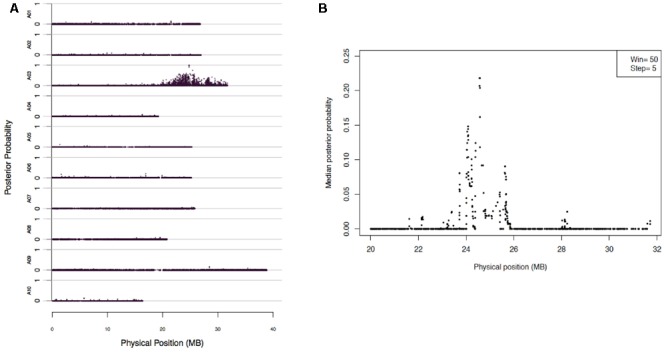
BSR-Seq to map Rcr2 according to the method described by Liu et al. (2012). **(A)** The physical position of each SNP marker (*x*-axis) was plotted versus the probability of each SNP marker being in complete linkage disequilibrium with the causal gene (*y*-axis). **(B)** Chromosome A03 was scanned by using a window containing 50 SNPs with a step size of five SNPs. Within each window, the median linkage probability obtained from a Bayesian BSA analysis across all the 50 SNPs was determined and was plotted against the middle physical position of the window.

Mapping of *Rcr2* were carried out using the 173,383 SNPs through BSR-Seq. The linkage probability of each SNP was plotted against its physical coordinate in the *B. rapa* reference genome. Each of the SNP markers that had a high probability of being linked to the *Rcr2* gene clustered on chromosome A03. No SNP markers with high linkage probability were observed on any other chromosomes. One significant peak was observed between 20 and 32 Mb of chromosome A03 (**Figure 2A**). To narrow down the interval within which *Rcr2* is located, the length of chromosome A03 was scanned using a window of a fixed number of SNPs (*N* = 50) and a step size of five SNPs. A strong peak, indicating a high probability of complete linkage disequilibrium with *Rcr2*, was observed at physical position 22–26 Mb of the *B. rapa* reference genome (**Figure 2B**).

### Fine Mapping of *Rcr2*

**FIGURE 3:**
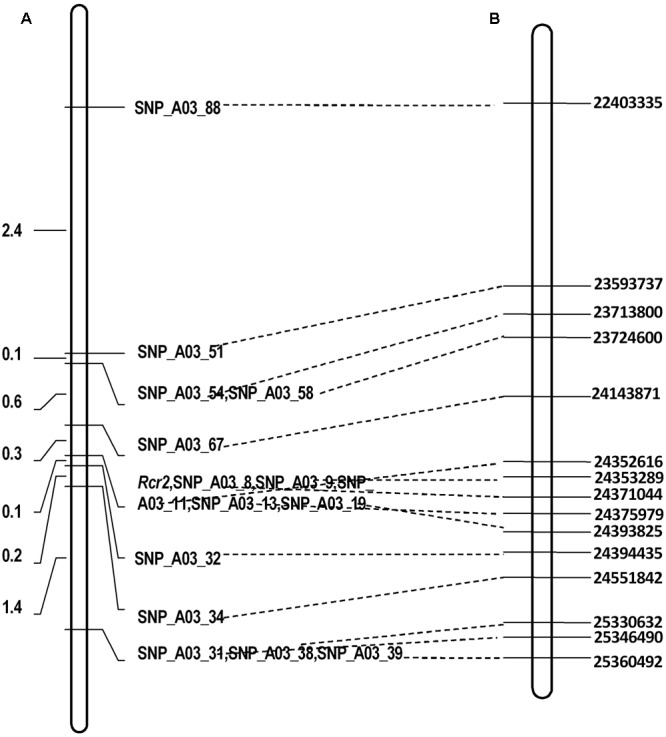
Linkage maps of the regions in which the *Rcr2* gene is located. Broken lines drawn regions defined by different molecular markers on *B. rapa* linkage group A03. **(A)** Fine mapping of *Rcr2* based on a F_1_ population consisting of 675 plants derived from ACDC × Jazz. The genetic distance is shown on the left. **(B)** Physical locations in bases (right) of the SNP markers.

There were 490 SNP sites identified at physical position 22–26 Mb of chromosome A03 where *Rcr2* was mapped using BSR-Seq (Supplementary Table S1). To fine map the gene, 675 plants were analyzed using the KASP technology, including 90 R and 90 S plants for RNA-Seq in the F_1_ segregating with 15 SNP markers (Supplementary Table S2). This analysis indicated that *Rcr2* was flanked by SNP_A03_32 and SNP_A03_67 at 0.1 and 0.3 cM from the markers, respectively, in an interval of 0.4 cM. Five SNP markers (SNP_A03_08, SNP_A03_09, SNP_A03_11, SNP_A03_13, SNP_A03_19) co-segregated with *Rcr2* (**Figure 3**).

### Identification of Variants in the Target Region

SNP_A03_32 is located in gene *Bra019406* at site 24,143,871 and SNP_A03_67 in gene *Bra038775* at site 24,394,435 (**Figure 3**). The physical distance between these two markers was 250,565 bases. There were 36 genes annotated in this region of reference genome v1.5.

The numbers of polymorphic variants (SNP and InDel) between the R and S bulks was assessed in the genes in the target region. Fourteen genes (*Bra038774, Bra038770, Bra038768, Bra038767, Bra038766, Bra038765, Bra038758, Bra038756, Bra038755, Bra038751, Bra038750, Bra038749, Bra019411*, and *Bra019408*) in the region did not show any expression and no short reads were assembled into the reference genome, so no variants could be identified in the genes. Similarly, eight genes (*Bra038773, Bra038772, Bra038769, Bra038763, Bra038759, Bra038748, Bra038747*, and *Bra019407*) had short reads with sequence counts <5 in either the R or S pooled sample, and number of poly variants <1 and so variants could not be identified. The remaining 14 genes had >5 sequence counts in both the R and S pooled samples, but the numbers of polymorphic variants differed among the genes (**Table 5**). Four genes (*Bra019409, Bra019410, Bra019412*, and *Bra019413*) encode TNL-class disease resistance proteins, assessed using BLASTX to *A. thaliana* and gene ontology annotation from http://brassicadb.org/brad/index.php (Supplementary Table S3).

**Table 5:** Number of polymorphic variants (SNPs and Indels) in the 14 genes located in the *Rcr2* interval that could be identified.

Gene	No. of variants			No. of short reads	
	SNP	Indels	Total	R	S
*Bra038775*	29	0	29	42	17
*Bra038771*	18	0	18	42	26
*Bra038764*	0	0	0	39	43
*Bra038762*	21	0	21	25	8
*Bra038761*	15	1	16	43	27
*Bra038760*	1	0	1	6	6
*Bra038757*	25	0	25	50	32
*Bra038754*	6	0	6	19	14
*Bra038753*	5	0	5	18	15
*Bra019413*	13	0	13	27	15
*Bra019412*	1	0	1	6	6
*Bra019410*	18	0	18	62	47
*Bra019409*	2	0	2	19	26
*Bra019406*	16	0	16	35	20

### Analysis of Variant Types in the TNL Genes

**FIGURE 4:**
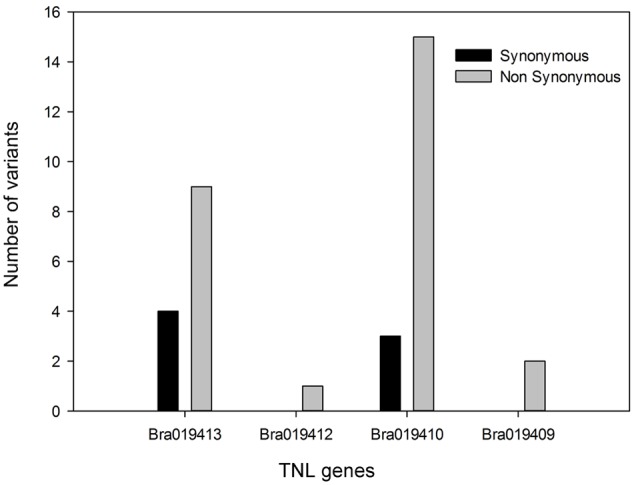
Number of unique poly variants in the four TIR-NBS-LRR genes from the pooled R samples.

In total, 34 synonymous and non-synonymous variants were identified from the coding sequences in the four TNL genes (Supplementary Table S4). Non-synonymous variants occurred in each of the TNL genes, but *Bra019410* and *Bra019413* carried more non-synonymous variants that uniquely occurred in the R samples than the other two TNL genes (**Figure 4**). Synonymous variants also occurred in *Bra019410* and *Bra019413* (**Figure 4**). No nonsense or frameshift variants were identified.

## Discussion

RNA-Seq is a robust approach for quantifying gene expression that has been widely used for transcriptome analysis in a range of organisms (Liu et al., 2012). It also can be used for identification of DNA variants. A BSR-Seq mapping strategy that relied on RNA-Seq data was used to map the *gl3* locus in maize (Liu et al., 2012). A similar strategy was used in the current study to map *Rcr2* into *B. rapa* chromosome A03 in an interval of 4 Mb region. Almost 500 SNP sites from the BSR-Seq were identified in the *Rcr2* interval, and 15 SNP markers were genotyped in the F_1_ segregating population (675 plants) using KASP assays. *Rcr2* was further narrowed into an interval of 0.251 Mb through linkage analysis. This demonstrates that BSR-Seq in combination with KASP analysis is a powerful approach for fine mapping of causal genes.

In Canada, five pathotypes of *P. brassicae* (pathotypes 2, 3, 5, 6, and 8) were initially identified based on the differential system of Williams (1966), with pathotype 3 the most prevalent on canola in the prairie region (Strelkov et al., 2006, 2007). Several canola cultivars were developed that exhibited strong resistance to these pathotypes (Strelkov et al., 2007, 2016). However, this resistance has been overcome recently by new strains of the pathogen (Strelkov et al., 2016). Both *Rcr2* donor (Chinese cabbage cv. “Jazz”) and *Rcr1* (pak choy cv. “Flower Nabana”) donor were highly resistant to pathotypes 2, 3, 5, 6, and 8 (Peng et al., 2014). However, the Chinese cabbage cv. “Jazz” was susceptible to the new strains of the pathogen while the pak choy cv. “Flower Nabana” was resistant (F. Yu and G. Peng, unpublished data). This indicated that *Rcr2* might confer a different resistance specificity from *Rcr1*. Another possibility was that pak choy cv. “Flower Nabana” carried additional gene(s) for resistance to the new strains. Clearly, this still needs to be determined.

*Rcr2* was initially mapped to chromosome A03 based on clubroot reaction to pathotype 3. However, donor cv. “Jazz” was also resistant to each of the other four pathotypes previously identified in Canada (Peng et al., 2014). To determine if *Rcr2* conferred resistance to the range of Canadian pathotypes, 46 F_2_ lines originating from 46 F_1_ plants were developed. Resistance to pathotypes 2, 3, 5, 6, and 8 was associated in each of the 46 lines (**Table 2**). This demonstrated that resistance to pathotypes 2, 5, 6, and 8 was significantly correlated with *Rcr2* and resistance to all of the original Canadian pathotypes of *P. brassicae* were controlled by the same gene or linked genes. Similar results were obtained from the F_2_ lines containing *Rcr1* (Yu et al., 2016) and BC_1_S_1_ lines containing *Rcr4* (Yu et al., 2017). A resistance gene on chromosome A08 in rutabaga (*B. napus* subsp. *napobrassica*) also conferred resistance to all five pathotypes (Hasan and Rahman, 2016).

In the current study, *Rcr2* was mapped in an interval of 0.4 cM, flanked by SNP markers SNP_A03_32 and SNP_A03_67 on *B. rapa* chromosome A03. Five markers (SNP_A03_19, SNP_A03_13, SNP_A03_09, SNP_A03_11, and SNP_A03_08) that reside in four genes (*Bra019406, Bra019410, Bra019412*, and *Bra019413*) in the target region were genotyped using the KASP method. Each of the markers co-segregated with *Rcr2*, indicating that they were completely linked to *Rcr2*. These SNP markers also co-segregated with *Rcr1* (Yu et al., 2016), so it is possible that *Rcr2* is co-localized with *Rcr1*.

Four genes in the *Rcr1*/*Rcr2* interval belong to TNL gene families. The DNA variant profiles for the TNL genes for *Rcr2* differ from those for *Rcr1* (Yu et al., 2016). No nonsense or frameshift variants were found in the TNL genes (**Figure 4**), but all four types of variants (synonymous, non-synonymous, nonsense, and frameshift) were identified in the genes associated with *Rcr1* (Yu et al., 2016). This could be associated with a lower depth of sequencing used in the current study relative to the depth used in the identification of *Rcr1*, such that the entire range of DNA variants in the *Rcr2* interval was not fully captured.

Based on the analysis of polymorphic variants in the genes in the current study, it is unlikely that the genes *Bra019409* and *Bra019412* are candidates for *Rcr2* because few polymorphic variants were identified in these genes. *Bra019410* and *Bra019413* carried higher number of variants, especially those that result in changes in amino acid sequence. They also carried higher number of synonymous variants, which can affect protein conformation and function (Sauna and Kimchi-Sarfaty, 2011). However, there were more polymorphic variants identified in *Bra019409* and *Bra019410* than in *Bra019412* and *Bra019413* in the *Rcr1* interval (Yu et al., 2016). *Rcr1*/*Rcr2* were mapped in the same interval as a known clubroot resistance gene, *CRa*. However, previous characterization of *CRa* (Ueno et al., 2012) did not determine how it was related to the TNL genes in the target region. The complete *CRa* coding sequence (GenBank: AB751517.1) consists of 4223 bp (Yu et al., 2016). A Blast search^3^ indicated that *CRa* is homologous to the TNL genes identified (especially *Bra019410*), but distinctly different to these genes (Yu et al., 2016). In addition, a QTL for clubroot resistance, *Rcr4*, has also been mapped to the *Rcr1*/*Rcr2*/*CRa* region (Yu et al., 2017). The TNL gene(s) that correspond with *Rcr2*, and the relationship of *Rcr2* with *Rcr1/Rcr4* or *CRa*, can only be identified conclusively after all of the genes have been cloned. Cloning could also be used to resolve the uncertainty of whether *Rcr2* confers resistance against several pathotypes or represents several tightly linked genes.

In total, 15 robust SNP markers were associated with *Rcr2*. These markers could provide an effective and robust basis for introgression of *Rcr2* into canola using MAS.

## Author Contributions

FY and GP conceived of the study; FY designed the experiments, analyzed data, designed KASP primers, and found SNP flanking sequences; ZH performed gene mapping and analysis of KASP markers; GP and KF provided parental lines. ZH, XL, AD, BG, and MM tested plants for resistance to clubroot; ZH and FY drafted the manuscript. All authors reviewed the manuscript and approved the final draft.

## Conflict of Interest Statement

The authors declare that the research was conducted in the absence of any commercial or financial relationships that could be construed as a potential conflict of interest.

## Footnotes

1 http://brassicadb.org/brad

2 http://www.lgcgroup.com/

3 http://brassicadb.org/brad/blastPage.php

## Supplementary Material

FIGURE S1 The distribution of DSI values to five pathotypes in the F_2_ population consisting of 46 lines derived from ACDC × Jazz.
