# Supplementary figures and images for: Fine Mapping of a Clubroot Resistance Gene in Chinese Cabbage Using SNP Markers Identified from Bulked Segregant RNA Sequencing
Source: Front Plant Sci. 2017 Aug 28;8:1448. doi: 10.3389/fpls.2017.01448 (PMC5581393; doi:10.3389/fpls.2017.01448)

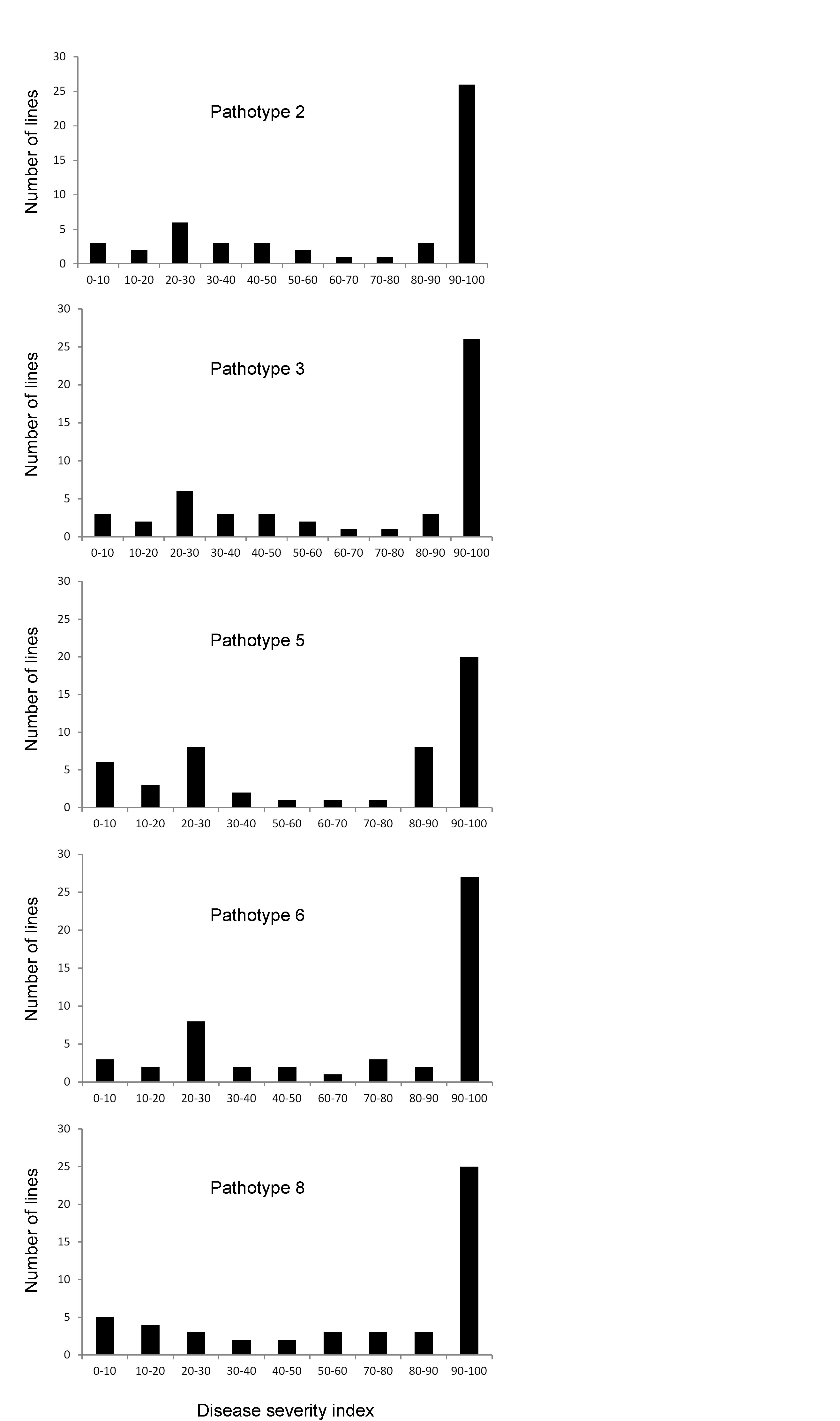

Supplement: FIGURE S1 — The distribution of DSI values to five pathotypes in the F2 population consisting of 46 lines derived from ACDC × Jazz. [file Image_1.JPEG]
