# Supplementary material for: Fine Mapping of a Clubroot Resistance Gene in Chinese Cabbage Using SNP Markers Identified from Bulked Segregant RNA Sequencing
Source: Front Plant Sci. 2017 Aug 28;8:1448. doi: 10.3389/fpls.2017.01448 (PMC5581393; doi:10.3389/fpls.2017.01448)
Supplement: Supplementary file 2 [file Table_1.DOCX]

**TABLE S1│ SNP sites identified between 22-26 Mb of chromosome A03**

| **Position** | **Reference allele** | **Alternative allele** |
| --- | --- | --- |
| 22003239 | A | T |
| 22004632 | A | G |
| 22004704 | G | A |
| 22004731 | A | C |
| 22005538 | G | A |
| 22005639 | A | G |
| 22023480 | A | T |
| 22115731 | A | G |
| 22123469 | A | T |
| 22123538 | T | C |
| 22124702 | A | G |
| 22228089 | C | T |
| 22228094 | C | T |
| 22228100 | G | A |
| 22228172 | G | T |
| 22228500 | T | C |
| 22228526 | C | T |
| 22229072 | T | G |
| 22270583 | A | G |
| 22292499 | G | T |
| 22311780 | C | A |
| 22312649 | A | G |
| 22325963 | C | A |
| 22325966 | T | A |
| 22326215 | A | G |
| 22326692 | T | C |
| 22326695 | A | C |
| 22326758 | C | T |
| 22389061 | G | A |
| 22402057 | G | A |
| 22402058 | A | C |
| 22402061 | G | C |
| 22402067 | G | T |
| 22402147 | G | C |
| 22402154 | C | A |
| 22555626 | T | C |
| 22589941 | A | G |
| 22592750 | T | C |
| 22593191 | T | G |
| 22593371 | T | C |
| 22593665 | G | T |
| 22593888 | T | C |
| 22593892 | A | T |
| 22711621 | T | A |
| 22713779 | T | C |
| 22714092 | G | T |
| 22721448 | A | G |
| 22833298 | C | T |
| 22835477 | G | T |
| 22835567 | C | T |
| 22867206 | A | G |
| 22867746 | T | A |
| 22867764 | T | C |
| 22867782 | T | G |
| 22867786 | G | C |
| 22878797 | C | T |
| 22883958 | T | C |
| 22900973 | T | C |
| 22927350 | T | A |
| 22927374 | T | C |
| 22934813 | G | T |
| 22950769 | T | C |
| 22950874 | T | C |
| 22983444 | C | A |
| 22984419 | T | G |
| 22987271 | A | G |
| 22998435 | G | A |
| 22998842 | T | C |
| 22998893 | G | A |
| 22998902 | G | A |
| 22999628 | C | T |
| 22999635 | C | A |
| 23041384 | T | C |
| 23041386 | C | T |
| 23046600 | T | A |
| 23070529 | A | G |
| 23073766 | T | G |
| 23167208 | G | T |
| 23167215 | T | A |
| 23167695 | A | T |
| 23168867 | G | A |
| 23168868 | T | C |
| 23205913 | A | G |
| 23206889 | T | A |
| 23208607 | A | G |
| 23208613 | A | G |
| 23208631 | T | A |
| 23208658 | G | A |
| 23208667 | G | T |
| 23209917 | C | T |
| 23209923 | T | C |
| 23228914 | T | A |
| 23271462 | C | A |
| 23272032 | T | C |
| 23273264 | G | A |
| 23273467 | A | T |
| 23273631 | A | C |
| 23273645 | C | G |
| 23273646 | G | C |
| 23273724 | T | A |
| 23335482 | C | T |
| 23368064 | G | C |
| 23368079 | G | A |
| 23368085 | G | A |
| 23368115 | T | C |
| 23368286 | T | A |
| 23399304 | G | T |
| 23399306 | C | G |
| 23399314 | T | C |
| 23399758 | G | A |
| 23458567 | A | C |
| 23464084 | A | G |
| 23464107 | G | A |
| 23464110 | G | A |
| 23464281 | A | G |
| 23464317 | T | C |
| 23464563 | C | A |
| 23465455 | T | G |
| 23466233 | T | G |
| 23507173 | A | C |
| 23507221 | A | G |
| 23528213 | T | C |
| 23528250 | C | T |
| 23528524 | C | T |
| 23557798 | A | G |
| 23578108 | G | T |
| 23578112 | C | A |
| 23579105 | G | A |
| 23579177 | G | C |
| 23579178 | C | G |
| 23593737 | G | A |
| 23605236 | G | A |
| 23612710 | A | T |
| 23613199 | A | G |
| 23613271 | T | C |
| 23614185 | T | C |
| 23614211 | C | T |
| 23648242 | A | G |
| 23649597 | A | G |
| 23721444 | A | T |
| 23721510 | A | G |
| 23727799 | G | T |
| 23728218 | G | C |
| 23730787 | G | T |
| 23730810 | G | C |
| 23730824 | C | G |
| 23731284 | G | A |
| 23731572 | A | G |
| 23731581 | C | T |
| 23731638 | A | T |
| 23731683 | T | A |
| 23731695 | T | C |
| 23731753 | G | A |
| 23731767 | A | T |
| 23731800 | T | C |
| 23732116 | G | A |
| 23732158 | T | C |
| 23732194 | G | T |
| 23733232 | G | C |
| 23733753 | T | C |
| 23733761 | T | G |
| 23733767 | T | C |
| 23733789 | A | C |
| 23733794 | G | A |
| 23733812 | C | G |
| 23792323 | G | C |
| 23792621 | G | T |
| 23792837 | C | T |
| 23792918 | G | C |
| 23792957 | G | A |
| 23794308 | G | A |
| 23794311 | A | G |
| 23848317 | C | G |
| 23947755 | C | G |
| 23959161 | C | T |
| 24009966 | T | A |
| 24009975 | T | C |
| 24009981 | C | A |
| 24009993 | T | C |
| 24019523 | C | T |
| 24019558 | C | T |
| 24019565 | T | C |
| 24019571 | T | C |
| 24019853 | C | T |
| 24019862 | G | T |
| 24020075 | T | C |
| 24020189 | G | C |
| 24020208 | G | T |
| 24020209 | C | G |
| 24020210 | T | G |
| 24020215 | T | A |
| 24020384 | A | T |
| 24020428 | C | T |
| 24074834 | G | C |
| 24075066 | T | A |
| 24075476 | G | T |
| 24075625 | T | C |
| 24080762 | A | G |
| 24080879 | T | G |
| 24083224 | A | G |
| 24083232 | T | G |
| 24083250 | T | C |
| 24083330 | T | A |
| 24083345 | T | C |
| 24083349 | A | T |
| 24083427 | T | C |
| 24083428 | A | G |
| 24084668 | A | C |
| 24084861 | C | T |
| 24085822 | T | C |
| 24086669 | T | A |
| 24086672 | T | G |
| 24086679 | G | A |
| 24086704 | G | A |
| 24086723 | C | G |
| 24086897 | G | T |
| 24086901 | C | A |
| 24087226 | A | G |
| 24087239 | A | G |
| 24088819 | A | C |
| 24089283 | G | T |
| 24090008 | T | G |
| 24091137 | G | A |
| 24091149 | C | T |
| 24091167 | C | T |
| 24091179 | C | T |
| 24091190 | T | A |
| 24091229 | G | T |
| 24143732 | C | T |
| 24143761 | C | G |
| 24143784 | T | G |
| 24143813 | A | G |
| 24143820 | C | T |
| 24143838 | G | A |
| 24143843 | C | A |
| 24143871 | G | A |
| 24144060 | G | T |
| 24145590 | A | G |
| 24145602 | T | A |
| 24145607 | A | G |
| 24146008 | C | G |
| 24146216 | C | T |
| 24192058 | G | C |
| 24192811 | G | T |
| 24192821 | C | A |
| 24225454 | A | T |
| 24225530 | G | T |
| 24226244 | G | T |
| 24226248 | C | T |
| 24227968 | G | T |
| 24228315 | C | A |
| 24228329 | T | A |
| 24229967 | A | G |
| 24248886 | C | A |
| 24248929 | C | T |
| 24248945 | C | T |
| 24248951 | C | G |
| 24248970 | C | A |
| 24248988 | T | C |
| 24253321 | G | C |
| 24256463 | C | A |
| 24257187 | G | C |
| 24278239 | A | G |
| 24278269 | C | G |
| 24278275 | G | A |
| 24278284 | C | T |
| 24278287 | A | C |
| 24278293 | G | T |
| 24278297 | C | T |
| 24278299 | G | A |
| 24278311 | C | A |
| 24278383 | C | T |
| 24290034 | C | G |
| 24290035 | T | C |
| 24290071 | G | A |
| 24290079 | G | A |
| 24290504 | T | C |
| 24292081 | C | T |
| 24292219 | G | A |
| 24351423 | T | C |
| 24352494 | G | A |
| 24352502 | T | C |
| 24352504 | G | A |
| 24352511 | T | C |
| 24352595 | A | G |
| 24352616 | C | A |
| 24352636 | T | C |
| 24352855 | C | T |
| 24353052 | C | T |
| 24353073 | A | G |
| 24353186 | G | T |
| 24353232 | C | T |
| 24353233 | C | T |
| 24353289 | T | A |
| 24371044 | A | T |
| 24371057 | C | A |
| 24371058 | T | A |
| 24374095 | A | G |
| 24374145 | A | C |
| 24374146 | T | G |
| 24374483 | C | T |
| 24374484 | G | A |
| 24374496 | G | T |
| 24374524 | A | C |
| 24374525 | T | G |
| 24374586 | G | C |
| 24374730 | A | G |
| 24374864 | C | G |
| 24374878 | G | T |
| 24374900 | C | G |
| 24374928 | G | A |
| 24374940 | C | A |
| 24374981 | G | C |
| 24375979 | T | C |
| 24378739 | A | G |
| 24378742 | G | A |
| 24378785 | C | G |
| 24378856 | T | C |
| 24378910 | A | G |
| 24381915 | T | C |
| 24381929 | C | T |
| 24393702 | G | A |
| 24393723 | T | C |
| 24393825 | T | A |
| 24393933 | T | C |
| 24393984 | T | C |
| 24394004 | G | T |
| 24394435 | T | G |
| 24551842 | G | A |
| 24556018 | A | T |
| 24560462 | C | T |
| 24560926 | G | C |
| 24572232 | C | T |
| 24572960 | C | T |
| 24574793 | G | T |
| 24574939 | C | T |
| 24575095 | T | C |
| 24575203 | A | G |
| 24576809 | A | G |
| 24576812 | C | T |
| 24576833 | T | C |
| 24576858 | A | C |
| 24576859 | T | C |
| 24576878 | T | C |
| 24576908 | A | C |
| 24576915 | T | C |
| 24577105 | A | G |
| 24578265 | C | T |
| 24578538 | G | T |
| 24578539 | C | T |
| 24578588 | T | G |
| 24645630 | G | C |
| 24723428 | C | T |
| 24723447 | A | T |
| 24724093 | C | T |
| 24724345 | T | A |
| 24724351 | T | G |
| 24724352 | G | A |
| 24724709 | C | T |
| 24724743 | G | A |
| 24726667 | T | G |
| 24726688 | C | T |
| 24726694 | T | C |
| 24726894 | G | T |
| 24787233 | C | T |
| 24789716 | C | A |
| 24790197 | C | T |
| 24799952 | C | T |
| 24799957 | G | A |
| 24799963 | A | C |
| 24800124 | A | G |
| 24800139 | T | G |
| 24800158 | G | A |
| 24800227 | A | T |
| 24800268 | G | A |
| 24800389 | T | A |
| 24800405 | T | T |
| 24809316 | T | C |
| 24811237 | T | G |
| 24864682 | G | A |
| 24949918 | G | A |
| 24950137 | A | G |
| 24950289 | A | C |
| 24950312 | T | A |
| 25058135 | C | T |
| 25120363 | G | C |
| 25137408 | T | A |
| 25274286 | A | G |
| 25319686 | G | A |
| 25330632 | A | C |
| 25346490 | A | G |
| 25330632 | A | C |
| 25346490 | A | G |
| 25356990 | T | A |
| 25360492 | G | A |
| 25397339 | G | A |
| 25360492 | G | A |
| 25412614 | A | G |
| 25414148 | C | T |
| 25414279 | A | G |
| 25422605 | C | T |
| 25422752 | T | G |
| 25422782 | G | A |
| 25435352 | C | A |
| 25435377 | C | T |
| 25435380 | A | G |
| 25435401 | T | C |
| 25435404 | A | G |
| 25435421 | A | G |
| 25435458 | C | G |
| 25435597 | C | T |
| 25435874 | G | A |
| 25447645 | G | A |
| 25474473 | G | A |
| 25474476 | T | C |
| 25474478 | G | A |
| 25474480 | A | G |
| 25533243 | T | A |
| 25632806 | A | G |
| 25632896 | T | G |
| 25632900 | C | T |
| 25632904 | G | T |
| 25632918 | T | C |
| 25632987 | A | G |
| 25633008 | G | A |
| 25633029 | A | T |
| 25633050 | G | T |
| 25633082 | A | C |
| 25647453 | T | C |
| 25649488 | T | C |
| 25649511 | T | C |
| 25649518 | G | C |
| 25649525 | T | C |
| 25649535 | T | G |
| 25650260 | G | A |
| 25650452 | C | A |
| 25650459 | G | A |
| 25650461 | C | A |
| 25650696 | A | G |
| 25650704 | C | T |
| 25650716 | T | C |
| 25650722 | A | C |
| 25650724 | A | G |
| 25650725 | G | A |
| 25657384 | C | T |
| 25661665 | T | G |
| 25667803 | G | C |
| 25668780 | T | C |
| 25668783 | A | T |
| 25670307 | C | T |
| 25670325 | G | T |
| 25670371 | T | G |
| 25670378 | A | G |
| 25670482 | C | T |
| 25670489 | C | T |
| 25670600 | A | G |
| 25670870 | A | C |
| 25671013 | G | A |
| 25671018 | C | T |
| 25671097 | T | C |
| 25671132 | T | G |
| 25671248 | G | T |
| 25671329 | A | G |
| 25671414 | G | A |
| 25671935 | T | C |
| 25697502 | C | T |
| 25702447 | A | T |
| 25705293 | A | G |
| 25706163 | A | C |
| 25706772 | G | T |
| 25706890 | C | T |
| 25714390 | A | G |
| 25723213 | G | A |
| 25723783 | C | G |
| 25724432 | C | T |
| 25724668 | C | T |
| 25724691 | G | A |
| 25743145 | C | T |
| 25743776 | G | T |
| 25743791 | A | G |
| 25743819 | G | A |
| 25744703 | T | G |
| 25760122 | A | G |
| 25836829 | T | G |
| 25838567 | T | C |
| 25838585 | G | C |
| 25838597 | A | G |
| 25839039 | T | C |
| 25839762 | C | G |
| 26028890 | A | G |

**TABLE S2│ KASP primer sequences in the *Rcr2* target region**

| SNP markers | Primers | Sequences (5'------3') |
| --- | --- | --- |
| SNP_A03_08 | SNP-A03_08Fam | GAGACAAAGCAGATGGAGTTGATGAA |
|  | SNP-A03_08Hex | GAGACAAAGCAGATGGAGTTGATGAT |
|  | SNP-A03_08Re | CCACCATCTTCTTTATCTTCTAGGTCTTT |
| SNP_A03_09 | SNP-A03_09Fam | AGAACTTGCCTGGGAGGTTACAAAA |
|  | SNP-A03_09Hex | AGAACTTGCCTGGGAGGTTACAAAT |
|  | SNP-A03_09Re | AGATCCCATAACCCTCAGTCCCAAA |
| SNP_A03_11 | SNP-A03_11Fam | AGTAAGCCATATCCTCACAGCATAG |
|  | SNP-A03_11Hex | CAGTAAGCCATATCCTCACAGCATAT |
|  | SNP-A03_1Re | GAGGGCAGACATGTCACGGGAT |
| SNP_A03_13 | SNP-A03_13Fam | ACACCCTTCCACAATTTCAAGCGT |
|  | SNP-A03_13Hex | CACCCTTCCACAATTTCAAGCGC |
|  | SNP-A03_13Re | CTTCATTTAGCTTGCTTTTTCAACGATGAA |
| SNP_A03_19 | SNP-A03_19Fam | AAAGCTCCAAACATCGTTCCCTTCA |
|  | SNP-A03_19Hex | AAAGCTCCAAACATCGTTCCCTTCT |
|  | SNP-A03_19Re | GTGAAGTGGAACCCCGTTGCGAA |
| SNP_A03_31 | SNP-A03_31Fam | ATCTTCGTTACCACTCAAATCCAGTA |
|  | SNP-A03_31Hex | CTTCGTTACCACTCAAATCCAGTG |
|  | SNP-A03_31Re | CCGAGAAAGGCATATACTTCTCCAGTT |
| SNP_A03_32 | SNP-A03_32Fam | AGCTCCTCAAAGTCTTCCACT |
|  | SNP-A03_32Hex | CTAGCTCCTCAAAGTCTTCCACG |
|  | SNP-A03_32Re | AACAGAGATTAGAGAGAAAGTAGATGTGAT |
| SNP_A03_34 | SNP-A03_34Fam | AACTTTAACGAACTGTCCATCAACG |
|  | SNP-A03_34Hex | GTAACTTTAACGAACTGTCCATCAACA |
|  | SNP-A03_34Re | CAAGAAGTATCCAGACGCTGAGCTT |
| SNP_A03_38 | SNP-A03_38Fam | AGTGGTTTTGTGATCAGCAATCTCT |
|  | SNP-A03_38Hex | AGTGGTTTTGTGATCAGCAATCTCG |
|  | SNP-A03_38Re | AAATGGCAAGGCTGAAGCAAGCCAA |
| SNP_A03_39 | SNP-A03_39Fam | AAGCCCAGCGAGGCCCAC |
|  | SNP-A03_39Hex | TAAGCCCAGCGAGGCCCAT |
|  | SNP-A03_39Re | GTCCAACAGACTCTCAGGCCCAT |
| SNP_A03_51 | SNP_A03_51Fam | GTTCTTTCCACAGTAAAGTCCACCAAG |
|  | SNP_A03_51Hex | GTTCTTTCCACAGTAAAGTCCACCAAA |
|  | SNP_A03_51Re | GCCCAAGACAGTCTTACTGATTCTGCT |
| SNP_A03_54 | SNP_A03_54Fam | CTTCGAGACGAAAACTGGTGTGGATG |
|  | SNP_A03_54Hex | CTTCGAGACGAAAACTGGTGTGGATC |
|  | SNP_A03_54Re | GTTTAGTCATCGCCCGGAACTGGTATCT |
| SNP_A03_58 | SNP_A03_58Fam | GGCTTTCAAAGGCCAGGAACCTTGAG |
|  | SNP_A03_58Hex | GGCTTTCAAAGGCCAGGAACCTTGAA |
|  | SNP_A03_58Re | CCAGACTCGTACATCCTTCAAGATCA |
| SNP_A03_67 | SNP_A03_67Fam | GGCAATCTATAAAGTCAGCGAAGCC |
|  | SNP_A03_67Hex | GGCAATCTATAAAGTCAGCGAAGCT |
|  | SNP_A03_67Re | GCAGATGTGAGCTCTCGGATCTCCTT |
| SNP_A03_88 | SNP_A03_88Fam | CTGACTCCAGCAGATAAGAGAAGTTTG |
|  | SNP_A03_88Hex | CTGACTCCAGCAGATAAGAGAAGTTTA |
|  | SNP_A03_88Re | GGTTTCACACATGATTGCATTTGAAGA |

Fam and Hex: Two SNP alleles were labelled with fluorescent tags Fam and Hex respectively; Re: Common reverse primer.

**TABLE S3│Best match to *Arabidopsis thaliana* genome for the genes in the *Rcr2* target region**

|  | |
| --- | --- |
| **Genes** | ***A. thaliana* Annotations** |
| Bra038775 | B160; B160; protein binding / transcription factor/ zinc ion binding |
| Bra038771 | SPHK1 (Sphingosine kinase1); D-erythro-sphingosine kinase/ diacylglycerol kinase/ sphinganine kinase |
| Bra038764 | CPL1, FRY2, ATCPL1; CPL1 (C-terminal domain phosphatase-like 1); double-stranded RNA binding / nucleotide phosphatase |
| Bra038762 | Pentatricopeptide (PPR) repeat-containing protein |
| Bra038761 | NRPB2, EMB1989, RPB2; NRPB2; DNA binding / DNA-directed RNA polymerase |
| Bra038760 | Unknown protein |
| Bra038757 | ATML1 (meristem layer 1); DNA binding / sequence-specific DNA binding / transcription factor |
| Bra038754 | TOM1, ATTOM1; TOM1 (Tobamovirus multiplication 1); protein binding |
| Bra038753 | MSRB2; MSRB2 (methionine sulfoxide reductase B 2); peptide-methionine-(S)-S-oxide reductase |
| Bra019413 | Disease resistance protein (TIR-NBS-LRR class), putative |
| Bra019412 | Disease resistance protein (TIR-NBS-LRR class), putative |
| Bra019410 | Disease resistance protein (TIR-NBS-LRR class), putative |
| Bra019409 | Disease resistance protein (TIR-NBS-LRR class), putative |
| Bra019406 | Apr3, Prh-26, Prh26, Atapr3; Apr3 (Aps reductase 3); adenylyl-sulfate reductase |
|  |  |
| Information was obtained from <http://brassicadb.org/brad/index.php> | |

**TABLE S4│ SNP sites identified in four TIR-NBS-LRR genes in *Rcr2* target region**

| **Gene** | **Physical position** | **Reference allele** | **Alternative allele** | **Impact** | **Developed markers** |
| --- | --- | --- | --- | --- | --- |
| Bra019413 | 24351423 | T | C | Synonymous | - |
| Bra019413 | 24352494 | G | A | Non Synonymous | - |
| Bra019413 | 24352502 | T | C | Synonymous | - |
| Bra019413 | 24352504 | G | A | Non Synonymous | - |
| Bra019413 | 24352511 | T | C | Synonymous | - |
| Bra019413 | 24352595 | A | G | Non Synonymous | - |
| Bra019413 | 24352616 | C | A | Synonymous | SNP_A03_11 |
| Bra019413 | 24352636 | T | C | Non Synonymous | - |
| Bra019413 | 24352855 | C | T | Non Synonymous | - |
| Bra019413 | 24353052 | C | T | Non Synonymous | - |
| Bra019413 | 24353073 | A | G | Non Synonymous | - |
| Bra019413 | 24353186 | G | T | Non Synonymous | - |
| Bra019413 | 24353289 | T | A | Non Synonymous | SNP_A03_08 |
| Bra019412 | 24371044 | A | T | Non Synonymous | SNP_A03_09 |
| Bra019410 | 24374095 | A | G | Non Synonymous | - |
| Bra019410 | 24374145 | A | C | Non Synonymous | - |
| Bra019410 | 24374146 | T | G | Non Synonymous | - |
| Bra019410 | 24374483 | C | T | Synonymous | - |
| Bra019410 | 24374586 | G | C | Non Synonymous | - |
| Bra019410 | 24374730 | A | G | Non Synonymous | - |
| Bra019410 | 24374864 | C | G | Non Synonymous | - |
| Bra019410 | 24374878 | G | T | Non Synonymous | - |
| Bra019410 | 24374900 | C | G | Synonymous | - |
| Bra019410 | 24374928 | G | A | Non Synonymous | - |
| Bra019410 | 24374940 | C | A | Non Synonymous | - |
| Bra019410 | 24374981 | G | C | Non Synonymous | - |
| Bra019410 | 24375979 | T | C | Synonymous | SNP_A03_13 |
| Bra019410 | 24378739 | A | G | Non Synonymous | - |
| Bra019410 | 24378742 | G | A | Non Synonymous | - |
| Bra019410 | 24378785 | C | G | Non Synonymous | - |
| Bra019410 | 24378856 | T | C | Non Synonymous | - |
| Bra019410 | 24378910 | A | G | Non Synonymous | - |
| Bra019409 | 24381915 | T | C | Non Synonymous | - |
| Bra019409 | 24381929 | C | T | Non Synonymous | - |
